# Rebooting “Failed” Family-Based Treatment

**DOI:** 10.3389/fpsyt.2020.00068

**Published:** 2020-03-05

**Authors:** Kellie R. Lavender

**Affiliations:** New Zealand Eating Disorders Clinic, Auckland, New Zealand

**Keywords:** family-based treatment, anorexia nervosa, adolescents, treatment fidelity, failed FBT, modified FBT

## Abstract

Family-based treatment (FBT) has become well established as the first-line evidence-based treatment for adolescents with anorexia nervosa. However, fidelity to the FBT model can be poor, and treatment is often augmented or modified in various untested forms in the hope of increasing its effectiveness and acceptability. The New Zealand Eating Disorders Clinic, a private specialist outpatient clinic in New Zealand, has been seeing increasing numbers of families presenting for treatment reporting an experience of “failed FBT”. All of the families who presented with a child under the age 19 living at home agreed to restart FBT with the author when re-engaging in treatment. This essay summarizes the experience of the author in repeating FBT with previously “failed” FBT cases over 20 months between 2017 and 2019. Common themes of the first course of FBT were identified that raised questions for the author as to whether FBT had been implemented with sufficient fidelity and proficiency the first time around. This clinical perspective essay describes how these identified issues were addressed when FBT was administered again. It does not intend to make broad claims, but instead is intended to be helpful to clinicians who are implementing FBT, to assist them in carefully examining and assessing whether key FBT principles and procedures have been exhausted before evaluating the need for modification or augmentation. Furthermore, this perspective provides suggestions as to how the identified common themes can be addressed if families re-present for FBT treatment after having had a course of “failed FBT”.

## Introduction

Manualized family-based treatment (FBT) is an empirically supported treatment for adolescents with anorexia nervosa with outcomes of full and sustained remission in 35–45% of cases (1–3). Full remission is defined as percent median body mass index (BMI) greater than 95% as expected for age and an Eating Disorder Examination (EDE) score within one standard deviation (SD) of the population mean (4). The efficacy data to date for manualized FBT are promising (5, 6). FBT has been established as a first-line treatment for adolescent anorexia, which is now reflected in treatment guidelines for eating disorders (7, 8). However, the effectiveness and acceptability of FBT in its manualized form has been questioned (9–11). There is also a growing trend among clinicians and treatment providers to describe the treatment they provide as “FBT informed” or “modified FBT,” although, to date, there is no definition of what these terms actually mean. Based on anecdotal discussions among colleagues and presentations at conferences, it seems that a “modified” approach entails some or all of the following: additional individual treatment for the adolescent, the use of a dietitian to provide dietary advice or meal plans, the use of adjunct groups (e.g., self-compassion, distress tolerance), or a planned hospital admission or residential stay to assist with weight restoration or management of eating disorder behaviors. Incorporating these kinds of interventions deviates from the empirically tested manualized version and is not informed by any research. There are indications that “modified” versions of FBT might even be more commonly practiced than the manualized version. In a study of 40 therapists providing treatment to youth with anorexia (12) concluded that there remained not one participant who reported practicing FBT with fidelity to the model. An online study examining FBT adherence demonstrated that one third of respondents deviated from the model (13). A study of therapist adherence to manualized FBT showed that adherence to the model decreased over time, and that adherence was strong only on behavioral interventions focused on meals and eating but weaker on other elements of the treatment such as modification of parental criticism and attending to general family process issues (14).

This perspective essay summarizes the author’s learning and reflections of providing FBT a second time around for cases with a reported history of “failed” FBT. Using the families’ narratives of their previous FBT treatment the author amalgamates identified issues into five key themes that appear to have contributed to the failure of the first course of FBT. Each of these themes will be discussed with a description of interventions of how these themes have been addressed in the second course of FBT.

## Summary of Case Data

Since this clinical perspective essay is based on reflections on a cohort of cases it is helpful to provide a summary of the key data of the cases. Nine cases in total presented to the New Zealand Eating Disorders Clinic over a 20-month period between October 2017 and May 2019, reporting “failed FBT”. All of them agreed to re-engage in FBT for a second time with the author. One family dropped out, four families are still in treatment, and four families have successfully completed treatment. The mean age at beginning of treatment was 17.3 years (range 14–19 years), with an average illness duration of 3 years (range 1–8 years). All are female with two cases with separated parents. Two cases identify as having 50% Maori descent. Seven cases have a diagnosis of anorexia nervosa restricting type, and one with anorexia nervosa binge-eating/purging type. Mean BMI at the beginning of treatment for all eight cases was 17.8 (range 16.6–19). Four cases have completed treatment after a mean of 21.75 sessions (range 17 to 25) with a mean BMI of 20 (range 18.5–21.6) and resumption of menses at EOT. Three completed an EDE-Q at EOT, all with a total score within one SD of community norms. The four cases still in treatment are also on track to achieve the same recovery standard, despite a longer duration of illness for two of the cases (>4 years). All of the cases are seen by the author, who is a certified FBT therapist and certified supervisor and has worked with eating disorders for 19 years and practicing FBT for 10 years. The author has regular specialist FBT supervision.

## Getting It Right From the Beginning

Theme one relates to whether families “started well”. All families reported knowing what FBT was; they had done their own reading about FBT; they had often connected with support groups and online forums. However, they reported mixed experiences of treatment delivery. In all cases, families were aware of the core tenets of FBT, namely externalization of the illness, parents being in charge of re-nourishment, being united, and not criticizing their child. In all cases, the entire family, including siblings, had been present at the first two sessions. It seemed that the structural components of the therapy had originally been set up appropriately. However, the *depth* of understanding and knowledge of these core principles and their translation into treatment appeared deficient. The parents reported that they felt they were prescribing something to their child in an inauthentic and mechanical way rather than embodying it. When session one was re-done, every family commented that they had not really “gotten it” the first time around. Particular attention was paid by the therapist to helping the parents *truly* understand the nature of anorexia and how it affected *their* child. Persistent circular questioning was used to draw out the parental understanding of the connection between what they knew about anorexia and its specific manifestations in their child. Multiple analogies and examples were re-visited until the therapist and family felt satisfied that the session one tasks had been fully addressed. Session two, the meal session, was a session that families wanted to skip because significant anxiety had been generated during the previous experience. The therapist needed to spend more time at the initial set-up exploring with sensitivity any reservations the families had and setting the right “tone”. This included empathy for the previous experience of the family and at the same time being mindful of the necessity of this session. It was framed up as a new learning opportunity, to assess where the anorexia may have taken ground; to supply the input needed to help set the family back on track, and to discover where unhelpful “blind spots” may have developed. In all cases, the intensity of the meal session was slightly reduced because the families had done it before. However, the participants had greater openness to learning because they “knew” the anorexia better. This provided an opportunity to empower the parents to utilize knowledge and experience they had already acquired in dealing with any problem areas that arose during the session.

## Facilitating Parental Empowerment

A strong early predictor of success in FBT is achievement of weight gain of 2.3 kg or more over the first month of treatment (15–17). In the reviewed cases, all patients had achieved weight gain of 2.3 kg or more in the initial stages of the first FBT treatment, which has been established by the above studies as a key predictor of increasing recovery chances. This raises the question, what happened? The parents described hearing the message clearly that weight gain was needed, and reported having been mobilized to ensure their child was eating sufficient amounts to gain weight. However, it seemed that the need to “re-feed” had become the *only* goal of FBT. The parents described the feeling of being in panic mode and managing to achieve early weight gain but eventually becoming stuck. Three families reported staying in phase one for up to 30 sessions and not being able to transition to phase two. Two families had transitioned to phase two on the recommendation of the previous therapist despite disagreeing. In two cases, at the suggestion of the therapist, when weight gain became stalled or distress remained high, the adolescents had received concurrent individual therapy, as an adjunct to FBT.

All parents, when seen for the second time, reported feeling exhausted, hopeless and sometimes feeling guilty about being a “failure” at parenting, or failing to feed their daughter. This situation called for particular attention. The issue of parental empowerment is a core tenet of FBT and one that is commonly misunderstood (18); yet it is the fundamental principle that underpins FBT. Empowerment is more than “being in charge of food,” which appeared to be the predominant understanding of the term. Parental empowerment is a complex concept, which refers to the process of parents becoming more confident in making decisions in the context of the tasks required to help their child recover from anorexia. Empowerment can only be achieved if the parents are on the same page, can understand the illness fully, and learn how to transfer their general parenting skills to the specific needs of treatment. If this is not achieved from the beginning and continually attended to throughout treatment, it can contribute to a number of problems in the long run. Problems in managing the anorexia behaviors that had been identified with the parents needed to be explored with the author for long enough to ensure that there was agreement and certainty about the plans and strategies the family were to implement. This also involved detailed and focused discussions when reviewing how plans had succeeded and what could have been done differently. Families are complex, and they do not have the knowledge or experience of anorexia at the beginning of treatment. The therapist needs to sensitively attend to issues such as parents not being in agreement or when one parent takes more control than the other. The therapist also needs to avoid being overly directive or too passive, thereby inhibiting the learning process for the parents. It is not easy to detect whether parents are not *really empowered*. This was one of the critical issues that required careful attention when undertaking FBT the second time. The empowerment of parents involves recognizing nuances of the delicate balance of actively identifying the family’s perspectives and strengths, reinforcing healthy decision making together, and simultaneously, setting clear expectations for treatment tasks and goals (18).

## Importance of Attending to Anorexic Behaviors, Not Just Weight Gain

In its first phase, FBT is highly focused on weight restoration (19). However, this does not occur at the expense of allowing anorexia behaviors and habits to go unchecked. All families reported that they had been instructed to keep a sustained focus on weight gain, and all families had achieved partial weight restoration the first time they did FBT. Families also reported that despite some weight restoration, eating disorder cognitions and body image concerns had remained unchanged. When this theme was explored further with the families, it became apparent that they had been under the impression that weight restoration was the *only* key to recovery, “As long as they eat and gain weight.” Families appeared to have an insufficient understanding of the need to challenge eating disorder-related behaviors. Instead, the families were offered individual treatment or a hospital admission for that purpose. Many of the adolescents admitted that they had hidden food, used water loading and weights, or secretly exercised throughout previous treatment. Families had not tackled fear foods or transitioned to normal levels of exercise before transferring responsibility back to the young person. The question of *why* cognitions were not changing had not been fully explored with families. This was a perplexing theme, as the FBT manual discusses the need in phase one as “Directing, redirecting and focusing the therapeutic discussion on food and eating behaviors and their management until food, eating and weight behaviors and concerns are relieved” (p.125, 19). Phase two involves gradual transfer of responsibility from the parents back to the child. The second time around, the first task the families had to learn was to take particular notice of anorexia behaviors, or uncover them, when the only cue was high distress and anxiety of their child. Parents also learned how to solve problems and how to extinguish *all* unwanted behaviors systematically. Parents reported that this was a new concept to them, a concept that had not been highlighted in their previous treatment, and, in some cases, had not even been mentioned. Frequently, these anorexia behaviors had become habit-based, and the adolescents and young adults were initially anxious about having to “give them up”. However, as the behaviors reduced over time, they were able to experience less anxiety and agitation with accompanying quieting of cognitions; the adolescents became very active participants during the later parts of phase two. Attending to the behaviors fully contributed to the affected adolescent feeling more “heard” and “understood,” and the experiments with parents became more collaborative as phase two progressed. Adolescents reported that in their previous treatment the focus had only been on increasing food amounts and that the lack of recognition by their parents and therapists of how distressing this was for them had increased their resistance to treatment and ultimately had resulted in their lack of faith and trust in their parents and in the effectiveness of FBT.

## Therapeutic Alliance and Compassion

A common theme raised by almost all of the parents was the concern about a lack of a therapeutic alliance between the previous therapists and their children. This is of interest because the therapeutic alliance in FBT has been demonstrated to be positive with parents and the adolescent (20, 21). In seven cases, the families had transitioned to individual treatment when FBT was “failing”. However, following this transition, every one of those seven patients deteriorated in weight and symptoms. The patients themselves later admitted that they had asked for individual treatment as a deliberate strategy to exclude their parents because they knew it would mean that there would be less pressure for weight gain and more chances of avoiding stress and conflicts around the challenges related to their eating behavior.

Re-booting FBT after the adolescent has already had individual treatment is always more challenging because of the reluctance of the young person to renounce their perceived control and autonomy. With five of the families who restarted FBT, this issue was addressed by agreeing that the young person would have more individual time at the beginning of sessions (the adolescents were older than 17 years of age). Initially, individual time was approximately 15 min (as opposed to 5–10 min), with sessions being 60–70 min in duration. However, it had to be made explicit that this was not “individual treatment” and the therapist was mindful of continuing to empower the parents, of the need to not be drawn into potentially divisive behaviors, and of reiterating the need for the parents to be part of conversations that involved making decisions. Particular attention was given, in a compassionate way, to linking the young person’s distress to being under the influence of anorexia and, at the same time, frequently acknowledging that it would be a normal instinctual response to want to avoid “feeling worse” by experiencing the intense anxiety when challenging the anorexia. This required the therapist to fully understand and believe that exposure was required to overcome these anorexia anxieties and to have a genuine, compassionate understanding for the affected adolescent and the parents, knowing how difficult it was to agree to decisions that would lead to having to tolerate distress and anxiety. Demonstrating compassion for the struggle of the adolescent is inherent to FBT and so is giving the adolescent more individual time as treatment progresses.

In all cases the second time around, there was never a question raised about the quality of the therapeutic alliance with the therapist. It is important for therapists to understand and feel confident that it is absolutely possible to maintain a strong therapeutic connection with the affected person without compromising the relationship with the parents and the fight against the anorexia. Although it is more challenging to restart FBT following individual treatment, it was useful that all families and adolescents acknowledged that the individual sessions had not resulted in the desired outcomes. This was helpful in directing the parents to the FBT framework without having to address requests for alternative forms of therapy at times of difficulties.

## The Importance of Full Completion of Treatment

None of the families had previously experienced phase three therefore, this phase was a new concept for them. Some families, the second time around, were tempted to finish treatment after the relief of weight recovery, having established normalized eating and exercise behavior. One study (2) suggested that phase three may not be needed for some families, but it was noted that cases with high levels of obsessive-compulsive features appeared to benefit from a more extended treatment regime. All patients and families discussed in this essay had already been in treatment for some time and understandably, were feeling exhausted and ready to move on. It took persistence on part of the therapist to ensure that families did not rush to finish or “jump over” the final stages. Typically, phase three is intended to be brief and is aimed at ensuring that the adolescent is on track developmentally. It also helps the family to identify areas of potential deficits that may have left their adolescent child vulnerable in the first instance (19). Most patients displayed heightened anxiety about “life without anorexia” and felt inept at knowing how to manage developmentally appropriate life challenges like moving away from home or navigating intimate relationships. In those cases, phase three was extended beyond the three to four sessions typically required. The parents also needed help to “let go” and time to experience that their child was genuinely managing well. In all cases, there was additional individual time at the beginning of the sessions. The content of these sessions no longer focused on eating disorder management or related concerns, but rather on other life issues to model age-appropriate developmental independence. The author does not see this as a modification to FBT but rather a reflection of the greater need, made more apparent by a longer duration of illness, to attend to ensuring the patient is developmentally “back on track”.

## Conclusions

There are several points worthy of reflection. The most critical one might be that it was possible to achieve full recovery using FBT treatment even after a course of previously “failed” FBT. It might be re-assuring to know for FBT therapists faced with similar cases that revisiting FBT is a valid treatment option. This perspective essay is based on eight cases seen by one therapist in one clinical setting over the course of 2 years. This raises the question of how often this phenomenon of families presenting for treatment as having “failed” may be occurring elsewhere. The themes discussed were present in all cases and, while comprehensive conclusions about the families’ previous treatment cannot be drawn, they were common and clear enough to question whether important key principles of FBT treatment had not been attended to with sufficient fidelity and proficiency the first time the families had engaged in FBT treatment. The emphasized points and solutions discussed in this essay are not outside what FBT clinicians should already be aware of. It might be helpful and reassuring to clinicians to know that with persistence, even a second course of FBT treatment can go well without the need for additional adjunct interventions like individual treatment. In fact, greater success was achieved in these cases by not adding major modifications of FBT. Even though this clinical perspective has not been based on a systematic review, the reflections on these cases warrant the message that, when an FBT case is not going well, deviating from the original FBT model needs to be carefully evaluated. Part of such an evaluation would need to be the clinician’s self-reflection on whether they have been delivering treatment with sufficient proficiency. All of the identified themes relate to key principles and tenets of FBT but their actual practical application might require some additional emphasis in teaching FBT. Clinicians may need to be better supported to develop sufficient depth of practice in FBT. An important step can be to ensure that clinicians receive adequate training and ongoing expert supervision including the need to focus on the themes addressed in this paper. It needs to be acknowledged that the narrative and the assumptions of the families’ previous experience is speculative and has not been systematically checked. Equally, the previous therapists may hold different views about the treatment they provided.

This clinical perspective has been written with the intention to assist practicing FBT clinicians with reflection and new learning. Further research would be helpful to help establish if these findings can be replicated when systematically investigated in a more extensive study.

## Author Contributions

KL is the sole author contributing to this paper and its associated contents.

## Conflict of Interest

The author declares that the research was conducted in the absence of any commercial or financial relationships that could be construed as a potential conflict of interest.
